# Reduced Histone H3 Acetylation in CD4^+^ T Lymphocytes: Potential Mechanism of Latent Autoimmune Diabetes in Adults

**DOI:** 10.1155/2015/285125

**Published:** 2015-12-29

**Authors:** Xi-yu Liu, Jiang-feng Xu

**Affiliations:** ^1^Department of Endocrinology, Fourth Affiliated Hospital of Zhejiang University School of Medicine, Yiwu, Zhejiang 322000, China; ^2^Department of Surgery, Fourth Affiliated Hospital of Zhejiang University School of Medicine, Yiwu, Zhejiang 322000, China

## Abstract

*Aims*. Latent autoimmune diabetes in adults (LADA) is the result of gene-environment interactions. Histone acetylation regulates gene expression and maybe interpret how environmental factors modify LADA. Hence, we studied the histone acetylation patterns in CD4^+^ T lymphocytes from LADA patients.* Methods*. Blood CD4^+^ T lymphocytes from 28 patients with LADA and 28 healthy controls were obtained to detect histone H3 acetylation and H4 acetylation. The gene expression of histone acetyltransferases (P300 and CREBBP) and histone deacetylases (HDAC1, HDAC2, and HDAC7) was measured by real-time polymerase chain reaction (RT-PCR).* Results*. Compared to healthy controls, reduced global H3 acetylation was observed in LADA patients' CD4^+^ T lymphocytes (*P* < 0.05). Global level of H4 acetylation was not statistically different. Among LADA, CD4^+^ T lymphocytes H3 acetylation was associated with glycosylated hemoglobin (HbA1c) and GADA titer. Compared to healthy controls, the expression of histone acetyltransferases CREBBP in LADA patients was downregulated, and the expression of histone deacetylases HDAC1 and HDAC7 was upregulated.* Conclusion*. A concerted downregulation of histone H3 acetylation was found in CD4^+^ T lymphocytes of LADA patients, and this might provide evidence of a novel epigenetic explanation for the pathogenesis of LADA and its complications.

## 1. Introduction

Latent autoimmune diabetes in adults (LADA) is a form of type 1 diabetes that occurs in adults, often with a slower course of onset. According to LADA China multicenter study, LADA prevalence in China is not low [1]. LADA is an autoimmune disease resulting from complex interactions between genetic and environmental factors. It is characterized by T-cell-mediated destruction of the insulin-producing islet *β*-cells. There are many immune cells involved in the pathogenesis of LADA, including CD4^+^ and CD8^+^ T lymphocytes, B lymphocytes, monocytes, and macrophages, and CD4^+^ T lymphocytes play a central role [2, 3]. Although the existing studies have provided much knowledge about genetic factors and autoimmunity associated with LADA [4, 5], the underlying mechanisms remain further studied.

Histone acetylation plays an essential role in regulating gene expression [6, 7]. Recent evidence indicates that histone acetylation contributes to the pathogenesis of some autoimmune diseases [8, 9]. And evidence suggests that diabetes and its complications also involve histone acetylation [10–12]. LADA is an autoimmune diabetes; we can speculate that histone acetylation is involved in the pathogenesis of LADA.

Type 1 diabetes is divided into two subtype-acute onset (classical type 1 diabetes) and latent onset (LADA). Compared to other autoimmune diseases, the research of histone acetylation in type 1 diabetes is relatively less and rare in LADA. Presently, Miao et al. performed research on histone modification in type 1 diabetes patients. Their study showed that H3K9 acetylation levels were markedly varied in type 1 diabetes monocytes relative to healthy controls [13], and monocyte H3K9 acetylation was significantly associated with the mean HbA1c level [14]. The object of their research was classical type 1 diabetes.

Classical type 1 diabetes characterizes islet function failure at onset. The present study of histone acetylation in type 1 diabetes patients focused on classical type 1 diabetes but rarely LADA. However, the islet function of LADA is not completely but partially destroyed, there is a certain time (several years often) interval that LADA progresses into depending on insulin treatment [15, 16]. Therefore, study and early intervention in LADA has a more important significance. The present study of histone acetylation in type 1 diabetes patients chose lymphocytes or monocytes, but CD4^+^ T lymphocytes play a central role in the pathogenesis of LADA, choosing CD4^+^ T lymphocytes to study is more direct and deep. In this study, we firstly investigated the global histone H3 acetylation and H4 acetylation status and the gene expression patterns of histone acetyltransferases and histone deacetylases which regulated histone acetylation in CD4^+^ T lymphocytes from LADA and healthy controls.

## 2. Research Design and Methods 

### 2.1. Human Subject Enrollment

**Figure 1:**
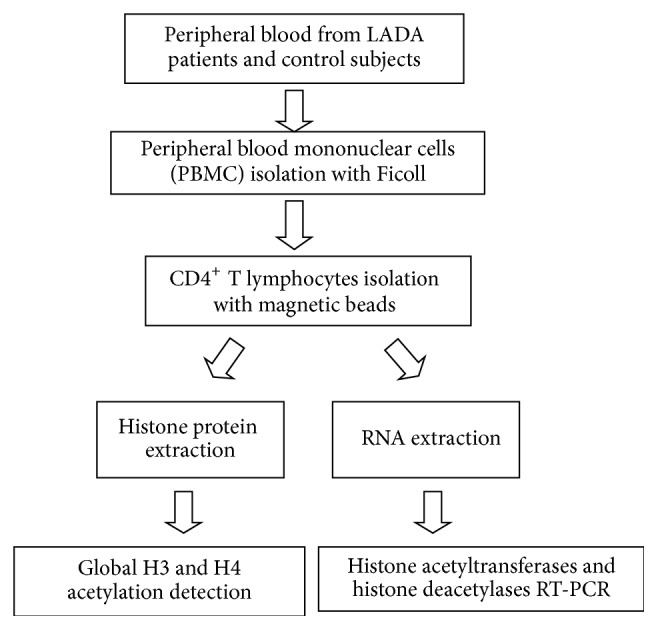
The study scheme. A total of 56 subjects (28 LADA patients; 28 healthy control subjects) participated in this study. Peripheral blood mononuclear cells (PBMC) were isolated from each subject with the Ficoll. CD4^+^ T lymphocytes were isolated by positive selection using magnetic beads. Then, Global H3 and H4 acetylation was detected in CD4^+^ T lymphocytes. The mRNA level of histone acetyltransferases gene (P300 and CREBBP) and histone demethylases gene (HDAC1, HDAC2, and HDAC7) was detected by RT-PCR.

This study had been performed with the approval of the ethics committee of the Fourth Affiliated Hospital of Zhejiang University School of Medicine and was in compliance with the Helsinki Declaration. Informed consent was signed by participants before their clinical records were used in this study. Patient records/information was anonymized and deidentified prior to analysis. We enrolled 56 volunteers into two groups, the first with 28 patients having a diagnosis of latent autoimmune diabetes in adults (LADA) and the second with 28 healthy controls. The diagnostic criteria of LADA proposed by the Immunology of Diabetes Society were used for this study. In this study, LADA was defined as patients aged above 30 years and who did not necessitate insulin administration for 6 months after the initial diagnosis and with glutamic acid decarboxylase autoantibody (GADA) positivity. All patients had no other autoimmune diseases. No history of autoimmunity and diabetes was reported in healthy control subjects; their OGTT value was normal and autoantibody status was not tested. The subject characteristics were shown in Table 1. There were no statistically significant differences between age or sex proportion in the two comparison groups.  Figure 1 demonstrated the experimental design for this study.

### 2.2. Isolation of CD4^+^ T Lymphocytes

A total of 60 mL of venous peripheral blood was collected from each patient and healthy control subject. Peripheral blood mononuclear cells (PBMC) were isolated by density gradient centrifugation using Ficoll gradients. CD4^+^ T lymphocytes were separated by positive selection using magnetic beads, according to the protocol provided by the manufacturer (Miltenyi Biotec, Bergisch Gladbach, Germany). The purity of CD4^+^ T cells was generally higher than 95%, as determined by flow cytometry.

### 2.3. Real-Time Quantitative PCRs

Total RNA was prepared by Trizol method (Invitrogen, USA); quantity and purity were determined by measuring optical density at 260 and 280 nm and stored at −80°C. Real-time quantitative PCR was performed using the one step SYBR PrimeScript RT-PCR kit according to the manufacturer's instructions (Takara). *β*-actin was used as an internal control. Primers of chromatin modifier genes were used, including the histone acetyltransferases (P300 and CREBBP) and the histone deacetylases (HDAC1, HDAC2, and HDAC7). Primer sequences were listed in Table 2.

### 2.4. Detection of Global H3/H4 Acetylation

Histone extraction and detection of global H3 acetylation and H4 acetylation were performed using the EpiQuik global histone H3 acetylation and H4 acetylation assay kits, according to the manufacturer's instructions (Epigentek). Briefly, cells were collected into a tube and used the provided lysis buffer to lyse cells. Use extraction buffer and TCA solution to extract histone. Histone proteins of samples and the acetylated H3 or H4 control were stably spotted on the strip wells. Add capture antibody after wash. The acetylated histone H3/H4 was recognized with high-affinity antibody. Add developing solution for developing color and then read absorbance on a microplate reader at 450 nm. The amount of H3/H4 acetylation was quantified by calculating OD (sample-blank)/OD (untreated control-blank).

### 2.5. Statistical Analysis

Results were presented as the mean ± SD. Group variables were compared by Student's *t*-test for continuous variable. Correlation was determined by Pearson's rank order or Spearman's rank order correlation, depending on the normality of the data. *P* < 0.05 were considered as significant. Real-time PCR data were analyzed by the comparative CT method (also named the 2^−ΔΔCT^ method), and the absolute of fold change > 2 was considered as significant.

## 3. Results

The subject characteristics were shown in Table 1. There were marked differences in diabetes duration and HbA1c between LADA patients and healthy controls (*P* < 0.01), and no significant differences in age, sex proportion, BMI, total cholesterol, triglyceride, HDL cholesterol, and serum creatine between the two groups (*P* > 0.05).

### 3.1. Global Histone H3/H4 Acetylation in CD4^+^ T Lymphocytes

**Figure 2:**
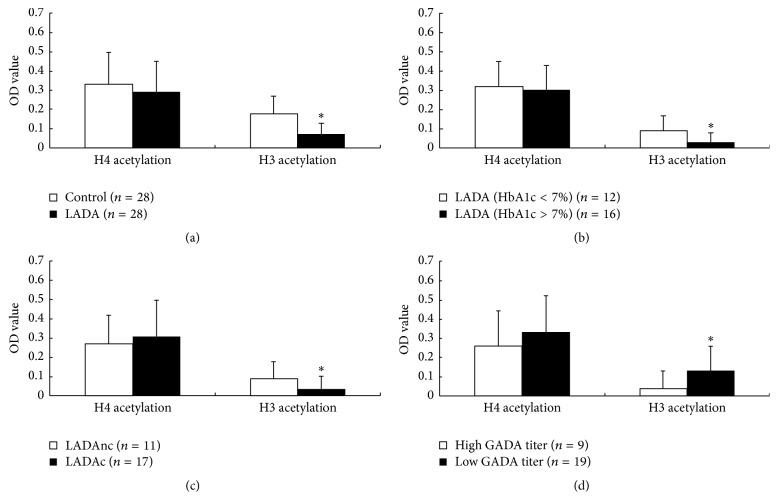
Global H3/H4 acetylation status in CD4^+^ T lymphocytes. (a) Global H3/H4 acetylation status in CD4^+^ T lymphocytes from patients with LADA (*n* = 28) and healthy controls (*n* = 28), ^*∗*^ *P* < 0.05, LADA patients versus healthy controls. (b) Global H3/H4 acetylation status in CD4^+^ T lymphocytes from patients with LADA, ^*∗*^ *P* < 0.05, LADA patients with HbA1c > 7% (*n* = 16) versus LADA patients with patients HbA1c < 7% (*n* = 12). (c) Global H3/H4 acetylation status in CD4^+^ T lymphocytes from patients with LADA, ^*∗*^ *P* < 0.05, LADA patients with complications (LADAc, *n* = 17) versus LADA patients without complications (LADAnc, *n* = 11). (d) Global H3/H4 acetylation status in CD4^+^ T lymphocytes from patients with LADA, ^*∗*^ *P* < 0.05, high GADA titer (*n* = 9) versus low GADA titer (*n* = 19).

To assess global histone H3/H4 acetylation levels in patients with LADA, we isolated CD4^+^ T lymphocytes from 28 LADA patients and 28 healthy controls. We found that reduced global H3 acetylation (*P* < 0.05) was observed in CD4^+^ T lymphocytes from LADA patients relative to healthy controls. H4 acetylation level showed no statistical difference between patients and controls (Figure 2(a)).

### 3.2. An Association between H3 Acetylation and HbA1c Was Visible

We next analyzed the relationship between H3 acetylation and HbA1c, and so forth. As shown in Table 3, we found that LADA CD4^+^ T lymphocytes H3 acetylation was related to HbA1c (*P* < 0.05). However, LADA patients' CD4^+^ T lymphocytes H3 acetylation was not related to fasting blood glucose, postprandial blood glucose, fasting C-peptide, postprandial C-peptide, age, duration of the disease, diabetic nephropathy, and cardiovascular disease.

When further dividing LADA patients group into two subgroups, HbA1c > 7% (*n* = 16) and HbA1c < 7% (*n* = 12), considering that the diabetes treatment goal was to achieve HbA1c < 7%. Compared to the LADA patients with HbA1c < 7%, reduced global H3 acetylation (*P* < 0.05) was observed in CD4^+^ T lymphocytes of LADA patients with HbA1c > 7% (Figure 2(b)).

As reported, HbA1c was associated with complications progression. We then divided the LADA patients group into two subgroups, those with complication (*n* = 17) and those without (*n* = 11). Compared to the LADA patients without complication, reduced global H3 acetylation (*P* < 0.05) was observed in CD4^+^ T lymphocytes of LADA patients with complication (Figure 2(c)).

### 3.3. An Association between H3 Acetylation and GADA Titer

LADA was an autoimmune disease, and GADA-positive was used to classify LADA. We compared the H3 acetylation lever between LADA patients with low GADA titer (18 units/mL ≤ GADA < 180 units/mL) and those with high GADA titer (GADA ≥ 180 units/mL). Compared to LADA patients with low GADA titer, reduced global H3 acetylation lever was observed in CD4^+^ T lymphocytes of LADA patients with high GADA titer (*P* < 0.05) (Figure 2(d)).

### 3.4. Histone Acetyltransferases and Histone Deacetylases Gene Expression in CD4^+^ T Lymphocytes

Because histone acetyltransferases and histone deacetylases regulate histone acetylation, in order to investigate the causes of reduced H3 acetylation patterns in LADA patients, we assessed mRNA levels of histone acetyltransferases and histone deacetylases genes in CD4^+^ T lymphocytes by real-time quantitative PCR.

**Figure 3:**
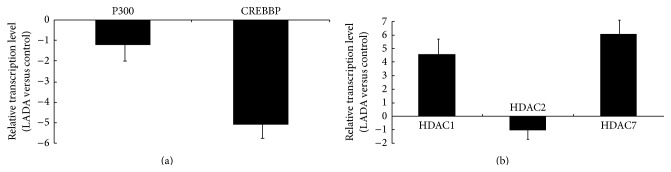
The mRNA levels of histone acetyltransferases and deacetylases in CD4^+^ T lymphocytes from patients and controls. (a) Relative mRNA levels of histone acetyltransferases (P300, CREBBP) in CD4^+^ T lymphocytes from patients with LADA (*n* = 28) and controls (*n* = 28), as measured by real-time PCR. Levels of target gene mRNA transcripts are normalized to *β*-actin. The absolute of fold change > 2 was considered significant, LADA patients versus healthy controls. (b) Relative mRNA levels of histone deacetylases (HDAC1, HDAC2, and HDAC7) in CD4^+^ T lymphocytes from patients with LADA (*n* = 28) and controls (*n* = 28), the absolute of fold change > 2 was considered significant, LADA patients versus healthy controls.

**Figure 4:**
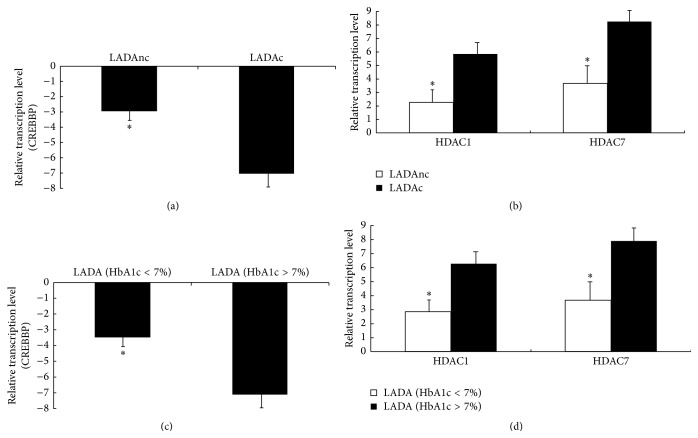
Association of complication and HbA1c with the expression of histone acetyltransferases and deacetylases in CD4^+^ T lymphocytes from LADA patients. (a) Relative mRNA levels of histone acetyltransferase (CREBBP) in CD4^+^ T lymphocytes from patients with LADA, LADA patients with complications (LADAc, *n* = 17) versus LADA patients without complications (LADAnc, *n* = 11), ^*∗*^ *P* < 0.05. (b) Relative mRNA levels of histone deacetylases (HDAC1 and HDAC7) in CD4^+^ T lymphocytes from patients with LADA, LADA patients with complications (LADAc) versus LADA patients without complications (LADAnc), ^*∗*^ *P* < 0.05. (c) Relative mRNA levels of histone acetyltransferases (CREBBP) in CD4^+^ T lymphocytes from patients with LADA, LADA patients with HbA1c > 7% (*n* = 16) versus LADA patients with patients HbA1c < 7% (*n* = 12), ^*∗*^ *P* < 0.05. (d) Relative mRNA levels of histone deacetylases (HDAC1 and HDAC7) in CD4^+^ T lymphocytes from LADA patients, LADA patients with HbA1c > 7% versus LADA patients with patients HbA1c < 7%, ^*∗*^ *P* < 0.05.

As shown in Figure 3, we found that the expression of histone acetyltransferase CREBBP in LADA patients was downregulated compared to healthy controls (Figure 3(a), the absolute of fold change > 2 was considered significant), and the expression of histone deacetylases HDAC1 and HDAC7 was upregulated (Figure 3(b)). Other histone acetyltransferases and deacetylases detected in our study showed no statistical difference between patients and healthy controls. Compared to LADA patients without complication, the expression of CREBBP in LADA patients with complications was downregulated (Figure 4(a)). Meantime, the expression of HDAC1 and HDAC7 was upregulated in LADA patients with complications (Figure 4(b)). Compared to the HbA1c < 7% LADA patients subgroup, the expression of CREBBP was downregulated and the expression of HDAC1 and HDAC7 was upregulated in LADA patients with HbA1c > 7% (Figures 4(c) and 4(d)).

## 4. Discussion

The objective of this study was to explore whether histone acetylation is involved in the pathogenesis and development of LADA. The reason for choosing the disease of LADA to study was as follows. The prevalence of diabetes increased year by year, and LADA is a subtype of diabetes of which molecular mechanism is unknown. Recently, histone acetylation is a hot research topic, and a large number of studies have shown that histone acetylation is associated with autoimmune diseases and diabetes; however, few studies focus on LADA. Hence, we studied the histone acetylation patterns in LADA patients. The rationale for choosing CD4^+^ T lymphocytes to study was as follows. Firstly, CD4^+^ T lymphocytes can be isolated from peripheral blood which could be obtained in a relatively noninvasive fashion during a regular visit. Next, the epigenome, unlike the genome, is cell type specific. Most importantly, LADA is characterized by T-cell-mediated autoimmune destruction of islets, and CD4^+^ T lymphocytes play a central role in the pathogenesis of LADA [2, 3]; therefore, choosing CD4^+^ T lymphocytes to study was more direct and deep.

We demonstrated the global histone acetylation pattern in CD4^+^ T lymphocytes from LADA patients. We found that reduced global H3 acetylation existed in CD4^+^ T lymphocytes from LADA patients and the reduced H3 acetylation lever was associated with GADA titer. LADA was an autoimmune disease, and GADA-positive was the most important autoimmune marker of LADA. We therefore speculated that H3 acetylation possibly contributed to the pathogenesis of LADA.

To investigate the causes of reduced H3 acetylation patterns in LADA patients, we assessed mRNA levels of histone modifier. The results showed that the expression of acetyltransferase CREBBP in LADA patients was downregulated and the expression of histone deacetylase HDAC1 and HDAC7 was upregulated. Histone acetylation was regulated by histone acetyltransferases and deacetylases [17, 18]. The low expression of the specific acetyltransferase CREBBP and high expression of deacetylase HDAC1 and HDAC7 may explain the dramatic reduction in global histone H3 acetylation which we observed in the CD4^+^ T lymphocytes from LADA patients.

We analyzed the relations with H3 acetylation and diabetic metabolic indexes. We found that H3 acetylation lever was associated with HbA1c. Meanwhile, we found relatively reduced global H3 acetylation was observed in LADA patients with complications compared to those without complication. The above two results were consistent with prior analyses from the DCCT [19, 20] showing a nonlinear relationship between HbA1c and complications progression. Miao et al. [14] found that type 1 diabetes monocyte H3K9 acetylation was significantly associated with the HbA1c, and the hyperacetylated promoters included over 50% genes related to the NF-*κ*B inflammatory pathway. Combined with our study, this suggested that the reduced histone H3 acetylation at multiple levels in the NF-*κ*B pathway or maybe other pathways could be related to the complication of LADA.

In conclusion, we found that global histone H3 acetylation level in CD4^+^ T lymphocytes from LADA patients was significantly reduced. This might provide evidence of a novel epigenetic explanation for the pathogenesis of LADA and its complications.

## Figures and Tables

**Table 1:** Subject characteristics.

Parameters	LADA patients (*n* = 28)	Control subjects (*n* = 28)	*P* value	LADA patients		
				Complication free	With complication	*P* value
				(*n* = 11)	(*n* = 17)	
Gender (male%)	46.4%	46.4%	1.0	45.6%	47.1%	0.79
Age (years)	43.0 ± 6.9	40.1 ± 6.3	0.82	39.3 ± 5.7	45.2 ± 6.9	0.25
BMI (kg/m^2^)	23.8 ± 3.2	23.2 ± 3.4	0.79	22.0 ± 1.1	24.8 ± 1.3	0.39
Diabetes duration (years)	9.7 ± 3.8	0	<0.01	6.8 ± 3.4	11.5 ± 4.7	0.03
HbA1c (%)	9.6 ± 2.5	5.1 ± 1.3	<0.01	7.8 ± 2.3	10.8 ± 3.1	0.04
FCP (nmol/L)	0.42 ± 0.26	—	—	0.57 ± 0.23	0.32 ± 0.28	0.12
Low FCP	8/28 (28.6%)	—	—	2/11 (18.2%)	6/17 (35.3%)	—
High titer GADA	(9/28) 30.0%	—	—	3/11 (27.3%)	6/17 (35.3%)	—
Low titer GADA	(19/28) 70.0%	—	—	8/11 (72.7%)	11/17 (64.7%)	—
Total cholesterol (mmol/L)	4.2 ± 0.9	4.3 ± 0.8	0.53	4.1 ± 0.6	4.3 ± 1.1	0.28
Triglyceride (mmol/L)	1.7 ± 0.5	1.8 ± 0.6	0.65	1.6 ± 0.4	1.7 ± 0.7	0.49
HDL cholesterol (mmol/L)	1.0 ± 0.3	1.2 ± 0.4	0.43	1.1 ± 0.3	0.9 ± 0.2	0.26
LDL cholesterol (mmol/L)	2.8 ± 0.6	2.9 ± 0.8	0.72	2.6 ± 0.7	2.9 ± 0.8	0.21
Serum creatine (*μ*mol/L)	102 ± 19.2	80 ± 11.2	0.09	90 ± 12.8	109 ± 16.7	0.12
Nephropathy (*n*)	8	0	—	0	8	—
Retinopathy (*n*)	5	0	—	0	5	—
Neuropathy (*n*)	8	0	—	0	8	—
Macrovascular complications	3	0	—	0	3	—

*n*: number of subjects; BMI: body mass index; FBS: fasting glucose; PBS: postprandial glucose; HbA1c: glycated hemoglobin; FCP: fasting C-peptide; PCP: postprandial C-peptide; HDL: high-density lipoprotein; LDL: low-density lipoprotein; GADA: glutamic acid decarboxylase antibody.

**Table 2:** Primer sequences used in real-time PCR.

Gene	Forward primer (5′-3′)	Reverse primer (5′-3′)
P300	CTGTATGTGCTCCAGAAC	GACAAAAAGGCAGTTCC
CREBBP	CTGCACACGACATGACT	GAAGTGGCATTCTGTTG
HDAC1	CAAGCTCCACATCAGTCCTTCC	TGCGGCAGCATTCTAAGGTT
HDAC2	AGTCAAGGAGGCGGCAAAA	TGCGGATTCTATGAGGCTTCA
HDAC7	CTTCTCCACAAGGACAAG	CTCCAGGGTTCTGTAGG
*β*-actin	GCACCA CAC CTT CTA CAA TGA GC	GGA TAG CACAGC CTG GATAGCAAC

**Table 3:** Correlation analysis of global histone H3/H4 acetylation in CD4^+^ T lymphocytes from LADA patients.

	H4 acetylation		H3 acetylation	
	(*n* = 28)		(*n* = 28)	
	*r*	*P*	*r*	*P*
FBS	0.015	0.860	−0.520	0.232
PBS	0.076	0.766	−0.159	0.553
HbA1c	−0.356	0.085	−0.623	0.021
FCP	0.445	0.079	0.117	0.843
PCP	0.242	0.525	0.154	0.762
Age	0.432	0.275	0.362	0.254
Duration of the disease	−0.153	0.161	−0.186	0.108
Diabetic nephropathy	−0.139	0.212	−0.264	0.279
CVD	−0.217	0.183	−0.287	0.163

LADA patients' CD4^+^ T lymphocytes H3 acetylation were not related with fasting blood glucose (FBS), postprandial blood glucose (PBS), fasting C-peptide (FCP), postprandial C-peptide (PCP), age, duration of the disease, diabetic nephropathy, and cardiovascular disease (CVD), but associated with glycosylated hemoglobin (HbA1c). H4 acetylation was not related to any of these factors.
